# The Reciprocal Insecurity Paradox: A Phenomenological Analysis of the Crisis Encounter Between the Mental Health Nurse and Service User With Complex Emotional Needs

**DOI:** 10.1111/nin.70125

**Published:** 2026-06-15

**Authors:** Michael Haslam, Mick McKeown, Emma Jones, Gary Lamph, Karen Wright

**Affiliations:** ^1^ School of Nursing and Midwifery University of Lancashire Preston UK; ^2^ School of Nursing and Midwifery Keele University Keele UK

**Keywords:** complex emotional needs, Crisis Resolution and Home Treatment, hermeneutic phenomenology, mental health nursing, ontological evasion, personality disorder, reciprocal insecurity, risk

## Abstract

While research is emerging, there remains an inadequate focus upon the experiences of those delivering and receiving care specifically for service users with complex emotional needs (CEN) within Crisis Resolution and Home Treatment (CRHT) settings. This study therefore utilised hermeneutic phenomenology to examine the lived experience of both those providing and receiving care within CRHT settings. Data were gathered via semi‐structured interviews with 14 participants (seven mental health nurses working within CRHT settings and seven service users with CEN who have received CRHT intervention). Transcripts were analysed using van Manen's hermeneutic phenomenological reflective method. A synthesis of participant accounts revealed several areas of convergence across the two groups, thus highlighting those ‘core structures’ of mental health crisis care as lived by participants in these encounters. Fears arising from structural vulnerabilities were illuminated as the *eidos* (or the ‘essence’) of the crisis encounter in CRHT settings, and at the heart of the crisis encounter, a central phenomenon of *reciprocal insecurity* is illuminated, whereby both parties' attempts to achieve a sense of personal safety inadvertently threatens that of the other. Findings highlight an urgent need for hermeneutic mediators; structures that provide the space and containment necessary for both parties to feel safe.

## 1 Background

Crisis Resolution and Home Treatment (CRHT) teams are multidisciplinary mental health teams, providing rapid assessment and intensive, short‐term treatment at home, as a direct alternative to inpatient admission. While mandated in the United Kingdom since 2000 (Department of Health 2000), comparable international models also exist within Norway, Australia, Germany and the United States (Johnson 2013). Yet, while a significant amount of literature already exists relating to the responses of healthcare staff towards individuals with complex emotional needs (CEN)^1^ and their presentation when in crisis, there is a paucity of recent research specifically relating to this issue within the context of CRHT settings (Haslam et al. 2024). Equally, where qualitative research studies have directly examined lived experiences of CRHT care (Nelson et al. 2016; Carpenter et al. 2013; Freeman et al. 2011) research is dated and typically addresses more generally the experiences of service users within these settings, rather than specifically focusing upon those with CEN.

Alongside earlier reviews concerning mental health crisis care for those with CEN (DeLeo et al. 2022; Warrender et al. 2021), our own 2024 qualitative evidence synthesis (Haslam et al. 2024) sought to address this research gap, offering insight into how CRHT was experienced both by those delivering and receiving care. More generally, our synthesis highlighted tensions between those delivering and receiving care. For instance, service user accounts focused upon the relational aspects of care received, thus highlighting the importance of this to their experience. Clinicians, meanwhile, focused upon those contextual issues linked to the management of organisational anxieties, expectations of others and organisational resources, all to the perceived detriment of personalised care.

Specifically, in relation to the care of people with CEN, our qualitative evidence synthesis also found that a focus upon risk alone or dismissing the person's perception of their own risk status, both undermined the person's need for individualised care and highlighted potential power differentials within the caring relationship. Furthermore, pejorative judgements linked to diagnostic labels led to exclusionary practices, mirroring those findings of earlier reviews (DeLeo et al. 2022; Warrender et al. 2021).

Phenomenologically, our qualitative evidence synthesis findings are understood through Merleau‐Ponty's (1945/2005) distinction between the *Körper* (the physical body as an anatomical object) and the *Leib* (the body as felt and ‘lived’). Where clinicians often defaulted to a focus upon the *Körper* through the prioritisation of individual risk or a diagnostic category, our recommendations advocated a shift in focus to embodied and relational working that prioritises the *Leib*. Within this context, this shift included the need for nurses to connect with the experience of the person in crisis.

Furthermore, our qualitative evidence synthesis (Haslam et al. 2024) corroborated the claim by Dalton‐Locke et al. (2021) that a solid evidence‐base regarding the impact of CRHT on service user experience was still lacking. More crucially, although research is emerging (Warrender 2024), there remains an inadequate focus upon the experiences of those delivering and receiving care specifically for service users with CEN within CRHT settings. Hence, this study explored these perspectives to better understand experiences, influential factors and impact.

## 2 Research Design

Phenomenology is a ‘powerful research strategy’ for investigating practice within healthcare (Neubauer et al. 2019, 90), as it is uniquely positioned to help people to learn from the experiences of others and thus chosen as the most suitable methodology for this research. Specifically, this study adopts a hermeneutic phenomenological approach (Heidegger 1927/1965). Data were analysed using van Manen's (1990) hermeneutic phenomenological reflective method, while the works of Merleau‐Ponty (1945/2005) and Oksala (2016) were also drawn upon to support a fuller interpretation of findings. Unlike traditional Husserlian phenomenology, however, which seeks a purely descriptive account of phenomena and ‘bracketing’ of researcher preconceptions, the hermeneutic approach taken here acknowledges both the interpretive role and the situatedness of M. H.; in this case, as a former crisis mental health nurse.

## 3 Methods

### 3.1 Sample Size and Recruitment

A purposive sample of 14 participants were recruited; a sample size which is aligned with phenomenological inquiry (Dibley et al. 2020; Smythe 2011; Crist and Tanner 2003). In keeping with a hermeneutic phenomenological approach, participants were recruited based on their lived experience rather than for representativeness (van Manen 1990). Hence, exclusively, all service user participants were women (despite this not being a criterion of inclusion/exclusion). This is perhaps reflective of an over‐representation of women; both working in the nursing profession, and those who either identify as having CEN or are ascribed associated diagnostic labels such as ‘borderline personality disorder’ (American Psychiatric Association 2013). Regardless, it was felt that findings still captured the essential structures of crisis care as delivered to those with CEN, although reflect a female‐gendered expression of this.

### 3.2 Mental Health Nurse Participants

Seven CRHT mental health nurse participants were recruited from different teams within a single NHS trust within the North of England. For this research study, age was not considered as important as years of service with CRHT, which ranged from just 2 to 9 years‐experience. All mental health nurse participants had previous nursing or healthcare experience prior to working for CRHT; variously working in health care assistant roles, inpatient services, the Acute Therapy Service and Community Mental Health Teams (CMHTs). This meant that participants had previous experience of working with people with CEN across a range of services before their CRHT role.

### 3.3 Service User Participants

Service user participants were recruited via professional networks and social media so were located across the United Kingdom (one residing in Scotland and the remainder in England). Location, however, was not considered to be an issue, given that the functional role of acute mental health crisis services is comparable across the United Kingdom, and the inclusion of participants from different regions was considered to increase the study's national relevance. Service user participants (who had had contact or treatment with CRHT services or their service equivalent) either self‐identified with a ‘personality disorder’ diagnostic label or had been diagnosed with this at some point in their lives.

### 3.4 Data Collection and Analysis

Qualitative data were collected between January 2024 and April 2025 via individual in‐depth semi‐structured interviews conducted by M.H. All participant interviews except one were conducted online. The remaining interview was conducted face‐to‐face, taking place at one of the CRHT bases at the participant's request. Once completed, interviews were transcribed verbatim and anonymous transcripts analysed by M.H. using Max van Manen's (1990) hermeneutic phenomenological reflection method (see Table 1), and findings were corroborated by the rest of the research team. Initially, data from the two participant groups were analysed separately, and then were synthesised to support an understanding of mental health crisis care for service users with CEN, as is lived from a dual nurse–user perspective.

**Table 1:** The six stages of Max van Manen's (1990) hermeneutic phenomenological reflection method.

Orientation to the phenomenon	Focusing upon the experience as is lived. Started from the findings of the literature review and the research question.
Investigating experience as this is lived	Participants' ‘lived experience’ was collected via semi‐structured interviews.
Hermeneutic phenomenological reflection	Reading and re‐reading transcripts while looking for areas of convergence. Data from each participant group were initially analysed separately. Mind‐mapping was also used to identify areas of convergence.
Describing the phenomenon through hermeneutic writing	Beyond just reporting findings, writing has also formed a part of the research itself. Through it, meaning was illuminated and ideas refined.
Maintaining a strong and orientated relation	Staying focused on the lived experience of the crisis encounter involved moving between the raw data, data analysis and the relevant literature. This required engagement with the *hermeneutic circle* (van Manen 1990; Gadamer 1989).^a^
Considering the parts and whole	Data from the two participant groups were synthesised here. Considering how specific ideas (such as the nurse's fear of the coroner's court, e.g.) contribute to the whole (fear as the overall essence of the crisis encounter).

^a^ Engaging with Gadamer's (1989) hermeneutic circle was essential here, considering the situatedness of M.H., as a former crisis practitioner. Given that the interpretative process involves what is commonly referred to as the ‘double hermeneutic’ (as in the researcher is making sense of participant accounts, while participants are in turn are making sense of their own experiences, Smith et al. 2009), engagement with the hermeneutic circle instead permits an ontological ‘fusion of horizons’.

### 3.5 Ethical Approval and Considerations

Ethical approval for this study was granted by HRA and Health and Care Research Wales (HCRW) (Approval Reference: 23/NW/0153). Participation in this research study was voluntary; all participants initiating contact to express their interest. Clear information and written consent forms were provided in advance of interviews, and acknowledging the potential vulnerabilities of service user participants, each was offered an opportunity in advance of the interview to verbally discuss participation, the potential for withdrawal and to ask further questions. Additionally, distress protocols were developed particularly for service user participants, although no recourse to these was necessary.

## 4 Findings

### 4.1 The Essential and Invariant Structures of the Crisis Experience

A synthesis of experiential accounts across all participants revealed several areas of convergence, thus highlighting those ‘core structures’ (van Manen 1990) of mental health crisis care, from the perspectives of both those delivering and receiving this. Despite the idiographic nature of hermeneutic phenomenology (as focusing upon participants' individual, unique and subjective experiences), the taking of an ‘interpretive leap’ here (Crowther and Thomson 2020; Smith et al. 2009), has moved our analysis beyond the simple description of individual experience as found in Husserlian phenomenology, to consider the implication for participants' way of ‘being‐in‐the‐world’ (Heidegger 1927/1965). Themes identified here therefore represent a synthesised interpretation of shared meaning; considered confirmable as they are explored alongside the existing evidence‐base, and transferable to others operating in similar contexts.

The four essential themes described below are considered to represent those invariant constituents of the mental health crisis encounter, at least for the participants in this research study. Collectively, they define the nature of mental health crisis care, illuminating unique dynamics experienced within the nurse–service user relationship. These themes are considered *essential* (van Manen 1990) given that they characterise the experience, and *invariant*, insofar as these are either explicitly reported or appear implicitly across all participant accounts:

- The fearful encounter
- The systemic squeeze
- Bureaucracy and disconnection
- The reified^2^ service user

**Figure 1:**
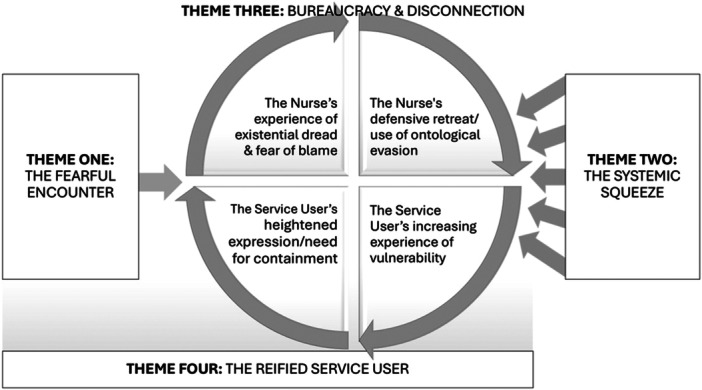
Reciprocal insecurity: highlighting the interplay between the four themes identified.

A diagrammatical representation of the four themes identified appears in Figure 1 and these are explained in more detail below. Throughout, the words of those used to illustrate ideas and representing key themes in this paper have been anonymised using pseudonyms derived from popular song titles. This decision humanises the findings, thus allowing readers to connect with the human experience behind the words; considered important given service user participants' discussion of their experiences of both dehumanisation and epistemic invalidation linked to diagnostic labels.

### 4.2 The Fearful Encounter

Linked to the phenomenological notion of embodiment, ‘the fearful encounter’ accounts for the initial dynamic between mental health nurses and service users, who both arrive at the encounter already experiencing anxiety and a state of existential fear. Service users' fears here are linked directly to their own embodied and visceral experiences of crisis and anxieties about what might become of them; Iris perfectly capturing this experience:

That loneliness, and the lack of control, and the free‐falling. Alice in Wonderland going down that hole…that's how it feels.

(Iris, SU)

Experiencing a mental health crisis as a lack of control was a sentiment mirrored across service user participant accounts:

Because you're not regulating your emotions in the way you need to… it becomes you can't control them.

(Ellie, SU)

In contrast, mental health nurses' fear was primarily driven by the potential for interpersonal conflict. Though understandable and perhaps predictable, this nevertheless provoked unease. Nurses here more generally talked about becoming the ‘brunt of abuse’ (Caroline, MHN) when the felt needs of service users were not met, and frequently expressed their frustrations around feeling ‘backed into a corner’ (Mandy, MHN). Helena (MHN) spoke about the difficult emotions arising from the ‘push and pull’ of this work, which impact upon the nurse–user relationship. The impact of this upon staff members was summarised by Caroline:

That hostility… From the offset, your back's up.

(Caroline, MHN)

Other mental health nurse fears related to professional risk (both linked to the fear of blame or of legislative processes where things might go awry). Such professional‐occupational insecurities were often associated with risk aversion; nurses talking about colleagues practising defensively, especially where there was the potential to lose their professional registration, or where there was a risk that nurses be held to account ‘in coroner's court’ (Michelle, MHN) for service failings.

Further, there was an existential risk to the ‘self’ as a competent nurse when working with those with CEN. Some nurses expressed self‐doubt in relation to their own skills. Even the most experienced nurses were not immune from such feelings:

It makes me feel as though I'm possibly not a good nurse, or that I'm not doing my job well enough… That I'm not doing enough for this person.

(Cecilia, MHN)

Where mental health nurse participants did express a personal sense of confidence or competence when working with those with CEN, they still described how colleagues, not necessarily within the team but across the whole service, lacked confidence. This was often attributed to a lack of experience and knowledge, although even intruded into questioning particular relational and communicative capabilities of colleagues.

Finally, for both service user and mental health nurse participants, fears were ameliorated where a reflective, supportive and non‐judgemental space was available for processing experiences:

Just safe space and just the feeling of feeling safe. It's like the biggest thing because it calms me down.

(Ellie, SU)

It's an open plan office. It's a safe place for people to express their frustrations. I'll get my negativity out afterwards if [the visit] doesn't go so well and knowing that that's there for you when you get back makes it a lot easier.

(Caroline, MHN)

Fears were exacerbated, however, where there was a lack of available structured interventions and clear pathways, particularly in respect of endings and transitions from the team as discussed below.

### 4.3 The Systemic Squeeze

The ‘systemic squeeze’ places the issues described above within their broader context, defining the environment in which fears associated with the crisis encounter are most acutely felt. First, the nature of the CRHT setting, designedly unique in that it is characterised by transient contacts and short‐term interventions, created a spatial and temporal void. Herein, the therapeutic relationships essential for emotional containment were more difficult to establish, insofar as they lacked the longitudinal constancy of those in other settings due to discontinuities of relationships:

[Trust] takes time to build and you don't have that, unfortunately. I get the nature of rotas and everything else but with home treatment teams, you're not always going to see the same person.

(Valerie, SU)

Visits were also restricted time‐wise, often meaning that assessments were hastily completed and interventions were limited. Time was further compromised by institutional demands for case‐data collection and appraisal of matters such as risk:

Sometimes when you have crisis intervention it's quite limited time‐wise and that means that they are firing risk assessment questions because they have to get through them.

(Iris, SU)

Inevitably, issues were compounded by the lack of consistency; not just involving inconsistencies in approach between different team members, but also the randomness of allocation of different personnel within teams, which limited connection and mutual comprehension. For instance, as explained by Mandy:

We've got so many people on the caseload they don't see the same people. So, you can't have that connection sometimes with them. It is [also] around the fact that we don't work seven days a week.

(Mandy, MHN)

Continuity was also an issue for mental health nurse participants who reported how the lack of clear structure and an ambiguity in their role increased anxieties. This was associated with different aspects of unpreparedness, or an absence of meaningful purpose being associated with discomforting feelings. ‘Just going around for a chat’ (Jo, MHN) was considered inadequate. A clear and structured plan, meanwhile, was felt to not just provide meaning and purpose to the visit but also alleviate the fears and increase the confidence of those visiting service users:

The reason structure's important is because it's a clear plan, isn't it, to some degree of what's going to be delivered? Patient knows where they stand. That's reducing that confusion, ambiguity.

(Caroline, MHN)

Second, endings and transitions of care were highlighted as a problem in respect of moving individuals out of the orbit of crisis care into the realm of ordinary community support. The CMHT was noted to reject referrals for fears of dependency, while the person still considered too complex for their General Practitioner (GP) instilled anxieties surrounding lack of ‘bridging’ support in relation to challenging needs beyond immediate crisis:

Nothing bridges the gap. That's the barrier that we've got. Nothing is in between, because a GP cannot deal with complex people.

(Mandy, MHN)

Without a clear discharge plan, or at least the means to implement this, nurses expressed a sense of therapeutic pessimism and described feeling ‘stuck’ (Mandy; Michelle, MHN). Holding on to people too long was against the grain of home treatment and only served to increase frustrations, especially if they were perceived as ‘exhausting’ (Michelle, MHN). A not unusual outcome of discharging someone without relevant follow‐up, however, was the prospect of them bouncing between inappropriate sources of support with people often ‘pushed from pillar to post’ (Jo, MHN). In starkly similar vocabularies, practitioners revealed their stress, contemplating the prospect of a revolving door and relative professional impotence to do anything about this:

‘What are we going to do with such and such a person? Where are they going to go?’ And then because you can't support them to access anything longer term, you end up discharging them knowing that they'll come back again.

(Helena, MHN)

Ultimately, the failure of the system to provide a sense of safety and ontological security for the mental health nurse means that, paradoxically, it can become a catalyst for the very risks it is designed to mitigate; further perpetuating a cycle of fear discussed below.

### 4.4 Bureaucracy and Disconnection

Grounded in the phenomenological dimension of relationality, the notion of ‘bureaucracy and disconnection’ highlights divergence between how nurses and service users interpret a sense of safety, and the ways in which they seek to achieve this. This theme illuminated a central tension between service users' need for relational safety and mental health nurses' reliance on bureaucratic tools that furnish an emotional distance and security needed.

First, for service user participants, safety was defined by the availability and ‘presence’ of the mental health nurse, which was central to the experience of feeling cared for. The feeling of being metaphorically ‘held’ was one which was repeated; for instance, such experiences described as ‘being in safer hands’ (Mary, SU), seeing the nurse as a ‘safety net’ (Iris, SU) and encounters as a ‘safe space’ (Ellie, SU). Where being ‘held’ occurred, it was felt to ‘lower the struggle a bit’ (Mary, SU). Again, this was powerfully articulated by Iris:

There's just something about, when I say ‘held’. It's an embrace. Like it's warmth. It's care. It's ‘we've got you’.

(Iris, SU)

In contrast, for some mental health nurse participants, bureaucratic tools served to moderate their own fears around the unpredictability of the other's crisis, providing a sense of control and security through ready‐made structures and objective measurements of personal and team effectiveness:

Sometimes it can help having both the objective, and an objective result there. So, we can also feed that back to the service user. This just shows how much improvement you've done.

(Michelle, MHN)

Furthermore, following national guidance on best practice (National Institute for Health and Care Excellence 2009, 2022), referring to the evidence‐base, and leaning into policy provided mental health nurses with a reassurance and a self‐confidence around decision‐making, especially within the context of risk:

I was following the evidence base… And I guess it just helps you with that self‐confidence and that self‐satisfaction as well.

(Jo, MHN)

Relying on evidence‐base and approved guidelines to justify intervention with service users also supported the management of service user and carer expectations; discussed unanimously across all mental health nurse participants. Done from the outset, this seemed to provide the mental health nurse with an additional sense of control, while reducing the likelihood of service users becoming ‘disgruntled’ (Helena, MHN). The ‘setting’ of realistic expectations (Jo; Helena, MHN) or ‘taking away’ of unrealistic expectations (Caroline, MHN) ensured that everyone was ‘on the same page’ (Helena, MHN). Mandy related this back to care planning:

We don't let them have high expectations because it's written. They discussed that on that care plan. They've chosen these three things. So, we'll do those three and when we finish, we'll move on.

(Mandy, MHN)

The review and completion of care plans and safety plans were seen by some as having utility within the crisis encounter, providing that they were ‘facilitative’ of collaborative discussions (Iris, SU). A predominant focus, however, on bureaucratic tools left nurses feeling they had ‘just done policies and procedures’ (Michelle, MHN) over any meaningful intervention. Meanwhile, an over‐reliance on tools such as risk assessments was experienced by some participants as an enactment of professional ‘detachment’ (Billie, SU); essentially removing mental health nurses' presence from the interaction. Some mental health nurse participants agreed:

Where's your eye contact when you're looking at an iPad? Where's your non‐verbals when you're looking at an iPad?

(Eileen, MHN)

This expression of the body‐object (*Körper*) approach was further supported where nurses indicated that standardised questions and checklists promoted an avoidance of delving too deeply for fear this would further heighten the individual's or even their own distress; either vicariously or in respect of the earlier remarked‐upon anxieties over professional capabilities:

When a person says something like that, we tend to not explore further… Obviously, that doesn't mean risk or anything like that. Risk is always explored. But their feelings. They're not always explored because we don't want to delve too much. Too much emphasis on that will heighten their already emotional state and they can play with it.

(Michelle, MHN)

Professional detachment, however, had the potential to increase service users' experiences of fear, heightening their need for containment. In the case of participants here, this manifested as increased expressions of distress. Within this context, service user participants talked about being ‘plunged into a bad darkness’ (Mary, SU) and ‘spiralling further’ (Billie, SU). For the nurse, this was perceived as the individual ‘upping the ante’ (Michelle; Cecelia, MHN); the negative connotations associated with this term stemming from its use within the context of the service user being reified as a diagnostic label or risk category as discussed below.

### 4.5 The Reified Service User

Where the previous theme explored the relational distance precipitated by professional fears and anxieties, ‘the reified service user’, meanwhile, examines the ontological *outcome* of this process. Central to the understanding of the key dynamic observed in the crisis encounter, this theme represents the dehumanisation experienced by service users when they perceive that their subjectivity is suspended, and the individual reduced to an object needing to be managed. In phenomenological terms, the notion of reification signals a collapse of both lived otherness (Relationality) and the lived body (Corporeality).^3^

For service users with CEN, reification appeared first as the mechanism underpinning the fearful encounter: the nurse often viewing the service user as a set of risks and diagnostic criteria prior to meeting with them. This process had already occurred where the nurse first interacted with a digital or paper representation of the service user, or at the point of handover from colleagues:

Often, you hear other colleagues saying, ‘oh, gosh, this person's like this and this person's like that’, and you're already a little bit anxious about what you might find.

(Helena, MHN)

Similarly, service user participants reported experiences of feeling reduced to a number on a caseload, as well as being defined by their ‘behaviours’ (Cassandra, SU) or their diagnosis. Visiting nurses ‘rushed in and rushed off’ in an attempt to ‘shake off’ the individual (Mary, SU). This provided further evidence that service users were reified as challenging, risky or anxiety‐provoking even prior to the encounter:

I felt very generalised. I felt I was just another number on their caseload. They weren't seeing me as a person. They just see you as this manipulative attention‐seeker, basically.

(Cassandra, SU)

This experience of reification further appeared as the mechanism through which ontological evasions occurred. As described in the previous theme, service user participants perceived the mental health nurse as withdrawing from the encounter when leaning into those standardised and procedural aspects of care, rather than seeking to understand the individual or gain an insight into their behaviours. Service user participants spoke of their experiences of those ‘are you still alive‐type conversations’ (Valerie, SU) with no scope for conversation outside of this, or of feeling reduced to ‘a set of paperwork’ (Cassandra, SU). Many participants such as Mary, reported how their interactions had been reduced to a safety‐orientated checklist:

I often feel a bit like sometimes ‘I'm coming to see you’, is a tick box exercise. They'll come in. They'll not always listen to what you're saying. They'll just sort of nod their head a bit and then they'll go. They do it to say they've done it, rather than really wanting to do it.

(Mary, SU)

The lack of embodied presence further heightened the service user's need for containment, manifesting as an increase in distress or risk‐taking, especially where service users felt the need to prove their distress:

I'd also say about not having to beg for something. Or not having to prove the distress I'm in. I think for a lot of people, self‐harm is reinforced by this. Proving distress. Worrying people.

(Iris, SU)

These heightened responses within the context of risk‐averse services served to provide confirmatory evidence of risk concerns linked to diagnostic categories, thus completing the reification of the service user as a diagnosis or risk object. Such a move further reinforced the nurse's instinct and rationale to manage potential harms through bureaucratic means and through body‐object (*Körper*) approaches.

### 4.6 Fear as the ‘eidos’ (‘Essence’) of the Crisis Encounter

At the core of this synthesis, fear and anxiety as arising from structural vulnerabilities were clearly highlighted. All participant accounts clearly pointed to numerous examples both of how the mental health nurse and service user first encounter the other, already experiencing heightened emotional states of fear and anxiety, and how care encounters continued to be framed by such. This suggests that, for the participants in this research study, fear was a shared *eidos* (or the ‘essence’) of a mental health crisis.

While maintaining the view of Merleau‐Ponty (1945/2005) that a complete eidetic reduction is impossible given our situatedness within the lived world, nonetheless, a reduction using Husserl's (1925/1977) free imaginary variation method was completed (see Table 2). This was not to assert an absolute truth, but to identify those necessary ontological structures that make a crisis encounter recognisable as such. This process was considered essential, especially where experiential accounts by some of the mental health nurse participants in this study indicated a *personal* confidence when working with people with CEN, and so on the surface, contradict the foregrounding of fear as the *eidos* of crisis work. The presence of personal confidence when working with people with CEN, however, does not necessarily debunk this idea but suggests a specific mode of attunement, thus merely refining our understanding of *how* fear remains eidetic to the crisis encounter.

**Table 2:** Fear as the ‘essence’ of the crisis encounter: An exercise in eidetic reduction.

Identifying the ‘regional essence’ Demarcating the fearful encounter from encounters experienced in other mental health settings	Temporal urgency: While acknowledging that the fearful encounter is not necessarily unique *per se* to CRHT settings, the intensity to which this is experienced is amplified. Fear is unmediated due to the acuity of the crisis.
	Lack of a relational buffer: Unlike long‐term teams (such as a CMHT), CRHT involves an element of ‘unfamiliarity’. Especially where there is a lack of history between worker and service user, there is a lack of relational buffer softening the initial fearful impact.
	The absence of a spatial container: Without the physical constraints of a ward, there is a lack of physical/institutional containment.
	The nurse as the ‘living boundary’: The lack of physical and temporal boundaries in CRHT settings means that the nurse's own body and presence become the sole structural support; the de facto source of containment, thus exposing the nurse to vulnerability. For both parties, this is what makes the fear felt so acutely, thus setting the scene for the fearful encounter.
The test of invariant structure Thus, confirming fear as the *eidos* or as ‘essential’ to the crisis encounter	Confidence as a moderator: Mental health nurse confidence does not necessarily disprove the presence of fear. Instead, confidence may be viewed as a moderator of, or a mode of ‘being‐with‐fear’.
	Confidence as a form of detachment: Data indicate that confidence is either an outcome of experience and learning to manage/contain personal anxieties, or conversely, may be a form of professional detachment. This indicates that fear is indeed a default state of the crisis interaction.
	The fearful service user: Regardless of whether the mental health nurse is experiencing a state of fear, the service user brings their own states of fear to the encounter, thus providing this essential component. Without this fear, the encounter dissolves into little more than a routine clinical interaction.

## 5 Discussion

### 5.1 Reciprocal Insecurity as the Relational Manifestation of Fear in the Crisis Encounter

Findings here are consistent with those of recent studies, confirming anxious and fearful mental health systems (Bond et al. 2025; Averill et al. 2024; Warrender 2024) whereby clinicians who experience a sense that their safety is challenged, become preoccupied with the avoidance, management and mitigation of risk and its associated outcomes. A narrow focus, however, upon risk management is said to result in the subjective sense for the patient of feeling unsafe (Averill et al. 2024). Furthermore, especially where patients are perceived as risky or challenging, they are likely viewed with diminished compassion (Bond et al. 2025), leading to acts and omissions that cause iatrogenic harm and further amplify service user insecurities (Warrender 2024). Such findings are well documented; sociologists such as Bauman (2000) having previously noted how insecurities grounded in uncertainty have become more or less baked into the practices of public services under neoliberal capitalism.

Hence, it is against this backdrop of institutional fear and defensive practice that we have identified *reciprocal insecurity*, a central phenomenon that is the relational manifestation of the fear experienced by both parties during the mental health crisis encounter. Reciprocal insecurity is the synthesis of the four themes identified, and is represented in Figure 1.

At its core are fearful people seeking to achieve a sense of safety. Data suggest, however, that each party's interpretation of safety, and the methods used to achieve this are not just *distinct* but also *conflict*; each party's pursuit of safety, paradoxically (and unintentionally) having the potential to bring forth a further discomfort in the other. A mismatch of intentionality is clear here between the service user's need for relational connection and distress relief, and the nurse's need for control (driven by the ‘systemic squeeze’). The sense of fear experienced here, therefore, appears to be neither the unique property of the service user or the nurse, but rather, a quality of the ‘in‐between’, or what Merleau‐Ponty (1945/2005) described as the *l'intermonde* (‘interworld’).

Linking these findings to our qualitative evidence synthesis (Haslam et al. 2024), which established a pattern of clinicians distancing themselves from service users, data here go beyond mere confirmation of a relational disconnect between nurse and service user. Instead, they suggest that its occurrence reflects shared (though *differently expressed*) experiences of fear. We are thus able to frame such professional distance (as in the mental health nurse's preoccupation with contextual or systemic issues over the relational encounter) as a professional defensive posture: a survival mechanism in response to the fears identified.

### 5.2 The Professional Defensive Posture as an Ontological Evasion

Arguably, insecurities remarked upon here likely reflect heightened uncertainties and responsibilisation for nurses; a manifestation of those broader systemic anxieties that are felt in their lived experiences of crisis care delivery. Sociologists working in a Foucauldian tradition have long emphasised the role of psychiatry and its dominant episteme in systems of governmentality; the practices of psychiatry serving a broader panoply of social control functions. Operating within an overarching risk society, the distribution of various hazards dictates the way society and government responses are organised, including those across healthcare systems (Beck 1992; Lupton 2023). This concern with risk, therefore, rather than seemingly more concrete phenomena such as dangerousness, generates insecurities that consolidate power within systems of governmentality (Castel 1991) while simultaneously (and unjustly) holding individuals responsible for systemic ills (Moth 2023), and precipitating further anxieties grounded in uncertainty (Bauman 2000). This is all evident alongside the appreciation that each may be held responsible for failures to avoid risks, further amplifying uncertainty and insecurity.

That said, while the retreat into bureaucratic and procedural‐based care established within these findings has a clear performative role (associated with the management and mitigation of clinical risk and legislative accountability), this might further be understood as an embodied response to being confronted with the other's (or even *one's own*) vulnerability. This is especially important in CRHT settings where the nurse's own body and presence are often the sole source of structural support (or a surrogate ‘psychic skin’ [Bick 1968]). Findings here might therefore be compared to those of Menzies Lyth (1988), who observed in the 1960s how the collective anxieties of staff in hospital settings were managed via a complex set of social defence systems which prioritised the needs of the institution and its workers over those of patients.

Here, checklist‐style assessment tools and lists of standardised questions in particular, were noted to lead to a collapse into body‐object (*Körper*) approaches, which, in a similar manner to those findings of Menzies Lyth (1988), appeared in some cases to constitute professional defensive postures, or ontological evasions (as in the refusal to fully inhabit the shared emotional and relational space of the service user experiencing crisis). Especially where the nurse was fearful, these allowed them to maintain a distance, thus not only evading the existential discomfort felt when confronted with another's suffering that they are unable to fix but also protecting the professional self from the chaos and emotional demands of the crisis encounter (Hochschild 1983). Alongside physical evasions, ontological evasions appear to serve as moderators of the nurses' anxiety and fear, facilitating an avoidance of the individual and the full burden of their distress‐as‐lived (*Leib*).

It is important to acknowledge here that bureaucratic measures, checklists and evidence‐based protocols are not, in themselves, inherently problematic. Rather their value is governed both by the nurse's intentionality and the ways and means by which they are communicatively worked with. Indeed, they were viewed more positively by both participant groups when used as facilitative tools to engender discussion around a person's needs, or where they reduced role ambiguity and reinforced nurse confidence. However, where the focus of the interaction was upon tool completion rather than the person and their needs (workers feeling compelled by systems to demonstrate that a risk assessment has taken place, rather than consider the assessment's purpose [Coffey et al. 2017]), they risked instead becoming a substitution for nurse ‘presence’. In this regard, record‐keeping concerning risk could tend towards fiction rather than reflecting a reality grounded in direct observation or dialogue between nurses and patients.

### 5.3 Reification as Arising From Diverging Epistemic Lifeworlds

Underpinning reciprocal insecurity appears to be the ontological process of reification, a concept that has application beyond the sociological critique of systemic ‘thing‐making’ (Lukács 1923/1971) and is also understood here as a phenomenological occurrence. As a mechanism through which professional distance within the crisis encounter is both established and understood, reification was observed most clearly in the findings where service user participants reported being reduced to their diagnostic category or risk status; both clinical abstractions, although treated with a concreteness that is usually reserved for objects to be managed. This likely occurred where risk dominated the mental health nurse's view (Felton et al. 2018). Reification both occurred a priori to the clinical encounter and was further reinforced by the nurse's defensive posture and resorting to body‐object (Körper) approaches.

Phenomenologically, notions of reification are aligned with the work of Toombs (1992) and Carel (2016), who provide a necessary framework for understanding how this manifests within clinical encounters. Reification is first reflected in what Toombs described as the ‘clinical gaze’, whereby the complex, situated, human subject (*Leib*) is stripped away, and illness reified as a tangible, localised and physical malfunction (*Körper*), as requiring standardisation and treatment. Within our findings, this was clearly illustrated where experiences of distress were viewed by the nurse as pathological, rather than as embodied and situated responses to challenging life circumstances and adversity. This event often occurred a priori to the clinical encounter and especially where service users were diagnosed with a personality disorder, itself an abstraction, although ‘defined’ by clusters of medical symptoms as though it were a real entity.

Second, reification is further reinforced through the dynamics of the encounter itself, specifically where the nurse and service user talk at cross‐purposes about such experiences (differences often stemming from the nurse and service user inhabiting different epistemic lifeworlds [Carel 2016; Toombs 1992]). Service users' subjective experience could be effectively sanitised; filtered out in favour of manageable symptomology, risk status and clinical categories. This typically leads to a sense of epistemic invalidation (Carel 2016; Fricker 2007), especially where the person, in their capacity as ‘knower’ is dismissed, or their accounts treated with an unwarranted disbelief. Again, clearly reflected in our findings: the detached professional's focus upon risk, policy and bureaucracy, despite this perhaps being *procedurally* correct, was experienced as *relationally* hollow. In failing to acknowledge the user's perspective, however, especially when this was in respect of perceived risk, this further prompted a need for service users to ‘prove’ their distress; their heightened expression of this provoking a reciprocal fearfulness, both providing further confirmatory evidence of risk to be managed, and solidifying the professional–client divide.

We suggest that reification is an intentional act: a way of perceiving the ‘other’ (in the phenomenological, Merleau‐Pontian^4^ sense). Whereas Toombs and Carel alternately understood reification as merely a failure to comprehend the patient's subjective world, our own findings allow us to both refine and expand further upon these ideas. For the mental health nurse, arguably, the reified service user is less an epistemic error than a functional necessity within the context of the crisis encounter, providing (and reinforcing) the professional distance required to manage those existential and professional fears inherent to mental health crisis work.

### 5.4 Situating the Crisis Encounter Within the Gendered Gaze

Returning to our earlier observation that our findings exclusively reflect a female‐gendered experience of the crisis encounter, it is also essential that the implications of this are considered: first in respect of the interpretation of data, and then also for the identified phenomenon of reciprocal insecurity. It is necessary, for instance, to acknowledge that the phenomenological texts utilised in this paper to interpret solely female‐coded expressions of distress and suffering were authored by men; their use, therefore, risking obscuring the gendered nature of power within the crisis encounter. Indeed, it would be remiss of us to also overlook the thinking of those feminist phenomenologists who have openly critiqued Merleau‐Pontian notions of the ‘lived body’ (*Leib*) as a neutral subject, arguing that bodies are in fact culturally moulded, shaped to correspond to normative expectations. Consequently, the focus upon the ‘lived body’ alone is too limited a framework to understand the gendered context of our findings (Oksala 2016).

Applying a feminist lens, therefore, to the context of this study, the reification of female service user participants (as a diagnostic label, for instance), might be interpreted as resultant of systemic power dynamics, inherent to both political and psychiatric systems. And at least for the women in this study, ‘upping the ante’ or the need to ‘prove’ distress, might otherwise be understood as less a clinical symptom than as acts of resistance against their experiences of epistemic invalidation. This is insofar as such experiences likely occur where subjective suffering is minimised or is even invisible under the patriarchal gaze (*Körper*). Thus, rendering this a structure of experience that is distinctively feminine.

Meanwhile, there is a necessity also to acknowledge here how the appearance of distress and a need for emotional containment might manifest differently for men with CEN who are experiencing crisis. Certainly, in the absence of male participants in this study, it becomes difficult to extrapolate specific behaviours to men during vulnerability, given that these are inevitably filtered through different socio‐cultural, gendered scripts (Oksala 2016). Nevertheless, when distinguishing between the universal structures of the crisis encounter, the underlying relational tensions at the core of reciprocal insecurity likely remain a constant, hence arguably extending this phenomenon also to men. While acknowledging that the very idea of gendered essentialism itself ought also to be treated with caution, further research is still required to establish whether a male‐gendered experience of crisis would reveal similar or distinct essential structures.

## 6 Implications and Recommendations for Practice

The identified phenomenon of reciprocal insecurity in this paper (and the processes of reification underpinning this) goes some way to resolving those previous fragmented understandings of the mental health crisis encounter. Issues here are less framed as resultant of personal failures. Aligning with the findings of Warrender's (2024) thesis (which identified the wider crisis system as being anxious and confused), the mental health nurse is re‐presented here as a compromised actor, working within a system that fails both parties.

Where the system does indeed fail to provide the adequate space needed for emotional security, we therefore recommend a need to enhance team structure to provide a safe, relational space where the nurse can first feel ‘held’ themselves, thus ‘containing the container’ (Bion 1962; Winnicott 1960). Where mental health nurse participants, for instance, reported positive interactions with service users, they also reported a felt sense of shared security and peer support within their teams; the CRHT office, for example, was maintained as a safe space where nurses were able to safely externalise fears, frustrations and the existential anxieties inherent to their work. With this in mind, we also propose that ‘hermeneutic mediators’, sense‐making processes such as regular reflective supervision, would enable the nurse to step outside of the fearful encounter and to interpret their situations from a broader perspective.

Further, remedial shifts in the nurse–user relationship are important, including the use of hermeneutic resources (Kidd et al. 2022), again aligned with the work of Carel (2016), which support the development of shared understandings around those experiences of illness that are difficult to articulate. Moving towards collaborative case formulation would actively resist the systemic pressure to resort to defensive posturing. This in turn supports a shift from viewing the service user as a reified risk object (*Körper*) back to a lived person (*Leib*).

## 7 Conclusion

Moving beyond a mere description of mutual experiences of crisis care, this research has exposed the ontological crisis sitting at the heart of the CRHT encounter. In establishing the notion that fear constitutes the *eidos* (or the ‘essence’) of this, at least for the participants in this study, our paper offers some insight into why relational dynamics within CRHT settings are sometimes difficult to navigate, thus going some way to resolving previously disparate and fragmented understandings of the mental health crisis encounter for individuals with CEN. We suggest that where this encounter fails, it is not necessarily due to individuals alone, but because the system propagates a state of reciprocal insecurity, whereby both parties' attempts to secure their own safety within the encounter, inadvertently (and paradoxically) threatens that of the other.

Building upon our 2024 qualitative evidence synthesis, these findings provide some critical food for thought, specifically around how the pursuit of professional safety using bureaucratic tools and through leaning into policy and the evidence‐base do not just serve a performative role. These can be considered ontological evasions, employed by the mental health nurse to escape those emotional demands of the encounter, especially where their bodies become the sole source of containment. The use of such body‐object (*Körper*) approaches, however, means that the service user is inevitably reified and their epistemic accounts invalidated, inadvertently contributing to their heightened distress. Thus, reinforcing a sense of fear and associated insecurities, which the system was ultimately designed to ameliorate.

Where reciprocal insecurity and the reified service user might be viewed as byproducts of compromised crisis systems, we have suggested that remedial action should be taken. There is a need, for instance, to prioritise those hermeneutic mediators which promote reflection and the relational connections essential to body‐subject (*Leib*) approaches. Such a shift is congruent with advocacy for a more general adoption of relational practice across mental health care (Haigh and Benefield 2024), wherein the findings discussed here can helpfully bolster the argument. Alongside this, we advocate safe spaces allowing nurses to feel ‘held’ themselves within their own teams. Indeed, we suggest that without these, the system itself will remain in a state of crisis, perpetually doomed by the mutual fear and distance that prevails on both sides of the checklist.

## Funding

The authors have nothing to report.

## Ethics Statement

Ethical approval for this study was granted by HRA and Health and Care Research Wales (HCRW) (Approval Reference: 23/NW/0153). In addition, approvals were granted by the University of Lancashire (Reference: HEALTH 01055).

## Conflicts of Interest

The authors declare no conflicts of interest.

## Declaration of Use of AI in Academic Writing

During the preparation of this manuscript, Gemini (Google) was used to manage text length and to review near‐final drafts for overall flow and coherence. All authors have critically reviewed, edited and revised the manuscript, and so take full responsibility for the accuracy, originality and integrity of the final published work.

## Data Availability

The data that support the findings of this study are available on request from the corresponding author. The data are not publicly available due to privacy or ethical restrictions.

## Footnotes

1 ‘Complex emotional needs’ (CEN) is a term used here to describe those difficulties and needs that are commonly (although not exclusively) associated with the more contentious and stigmatising (Bolton et al. 2014) diagnostic label of ‘personality disorder’ (World Health Organisation 2019; American Psychiatric Association 2013). While acknowledging that this term also has its critics (Porter et al. 2025), it is adopted here in a response to the ongoing debate around terminology (Sheridan Rains et al. 2021) and as one which may be more acceptable to some users of mental health services.

2 We suggest that a loss of the subject in this theme resonates with Lukács (1923/1971) notion of reification and *misplaced concreteness* (reification's literal meaning, ‘thing‐making’), as referring to the treatment of abstract ideas and people as if they were a single, tangible, fixed and object‐like entity. Such an abstraction functions to divorce the object from human action, thus situating it as an impersonal, and seemingly concrete ‘thing’ requiring standardisation and management.

3 This relates to the four ‘lifeworld existentials’ (van Manen 1990) used to guide reflection in phenomenological research. They are as follows: temporality (lived time, relating to subjective, rather than objective ‘clock’ time); spatiality (lived ‘felt’ space); corporeality (lived body, as referring to how individuals experience their world through their bodies), and relationality (lived relations, as relating to the interpersonal world we share with others).

4 According to Merleau‐Ponty, consciousness is always ‘directed towards a certain existing or possible task’ (Merleau‐Ponty 1945/2005, 114). This refers to *operative intentionality*: a pre‐reflective, embodied response to a situation that precedes objective thought.
